# New insights and novel perspectives in bileaflet mechanical heart valve prostheses thromboresistance

**DOI:** 10.1186/s13019-024-02786-9

**Published:** 2024-05-17

**Authors:** Giorgio Vigano, Sudip Shyam, Sushanta K. Mitra, Daniël K. M. Pollack, Massimo A. Mariani

**Affiliations:** 1 Department of Cardiothoracic Surgery, Heart Centre, University of Groningen, University Medical Centre Groningen, P.O. Box 30.001, Groningen, 9700 RB The Netherlands; 2 Micro & Nano-Scale Transport Laboratory, Waterloo Institute for Nanotechnology, Department of Mechanical and Mechatronics Engineering, University of Waterloo, Waterloo, Waterloo, ON N2L 3G1 Canada

**Keywords:** Warfarin-free, Mechanical heart valve, Nano-technology, Superhydrophobic, Thromboresistance

## Abstract

**Background:**

Although well-known for their thromboresistance, bileaflet mechanical heart valves (BMHV) require lifelong anti-thrombotic therapy. This must be associated with a certain level of thrombogenicity. Since both thromboresistance and thrombogenicity are explained by the blood-artificial surface or liquid-solid interactions, the aim of the present study was to explore BMHV thromboresistance from new perspectives. The wettability of BMHV pyrolytic carbon (PyC) occluders was investigated in under-liquid conditions. The submerged BMHV wettability clarifies the mechanisms involved in the thromboresistance.

**Methods:**

The PyC occluders of a SJM Regent™ BMHV were previously laser irradiated, to create a surface hierarchical nano-texture, featuring three nano-configurations. Additionally, four PyC occluders of standard BMHV (Carbomedics, SJM Regent^™^, Bicarbon^™^, On-X^®^), were investigated. All occluders were evaluated in under-liquid configuration, with silicon oil used as the working droplet, while water, simulating blood, was used as the surrounding liquid. The under-liquid droplet-substrate wetting interactions were analyzed using contact angle goniometry.

**Results:**

All the standard occluders showed very low contact angle, reflecting a pronounced affinity for non-polar molecules. No receding of the contact line could be observed for the untreated occluders. The smallest static contact angle of around 61° could be observed for On-X^®^ valve (the only valve made of full PyC). The laser-treated occluders strongly repelled oil in underwater conditions. A drastic change in their wetting behaviour was observed depending on the surrounding fluid, displaying a hydrophobic behaviour in the presence of air (as the surrounding medium), and showing instead a hydrophilic nature, when surrounded by water.

**Conclusions:**

BMHV “fear” water and blood. The intrinsic affinity of BMHV for nonpolar fluids can be translated into a tendency to repel polar fluids, such as water and blood. The blood-artificial surface interaction in BMHV is minimized. The contact between blood and BMHV surface is drastically reduced by polar-nonpolar Van der Waals forces. The “hydro/bloodphobia” of BMHV is intrinsically related to their chemical composition and their surface energy, thus their material: PyC indeed. Pertaining to thromboresistance, the surface roughness does not play a significant role. Instead, the thromboresistance of BMHV lies in molecular interactions. BMHV wettability can be tuned by altering the surface interface, by means of nanotechnology.

**Supplementary Information:**

The online version contains supplementary material available at 10.1186/s13019-024-02786-9.

## Background

**Fig. 1:**
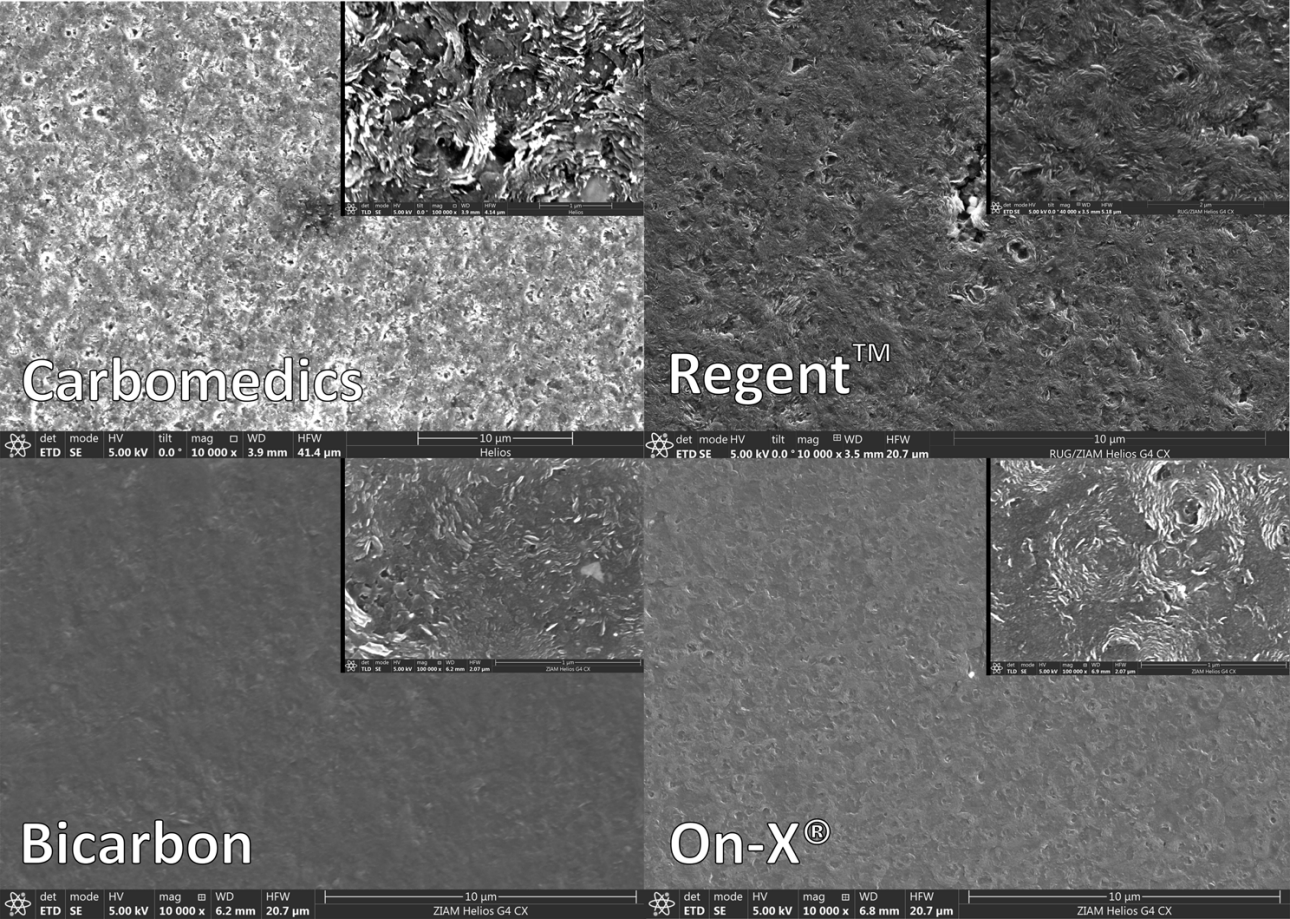
Scanning electron microscope (SEM) surface morphology of the untreated occluders: Corcym Carbomedics^™^ (Corcym SRL), St Jude Medical Regent^™^ (St Jude Medical, Inc, St Paul, Minn), Corcym Bicarbon (Corcym SRL), and On-X^®^ (On-X^®^ Life Technologies, Inc.). Some irregularities or imperfections of a scale of 5 μm can be seen

In recent years, biological heart valve prostheses have drawn the research interest over mechanical valves [3], although the long-term durability of biological heart valves is still a concern [1, 2]. In this regard, bileaflet mechanical heart valve prostheses (BMHV) deserves a renewed interest, particularly in light of the survival benefit associated with mechanical valves, that has been recently shown in young patients [4, 5]. Although closer and closer to the ideal replacement valve envisioned decades ago by Dr. Dwight Harken, presently available mechanical valves still require lifelong warfarin-based anticoagulation therapy [6]. All commercially available BMHV are based on concepts that remained unchanged for decades and share, to a similar extent, the same leading concept [2]. The treatment of BMHV surface focuses on the creation of an ideal solid, i.e., smooth, mirror-polished surface, in order to minimize the frictions between the fluid (blood) and the solid (valve), leveraging the low-friction principle (superlubricity). Nevertheless, microscopic analysis of the surfaces of mechanical heart valve revealed the presence of significant irregularities (Fig. 1) at a scale of 5 μm, which impact cell adhesion and thrombogenesis [7]. Besides, in order to explain the blood-artificial surface interactions, it seems reasonable to investigate the effective interaction between a blood droplet and the valve surface, as a liquid-solid wetting interaction.

In general, when a liquid droplet comes in contact with a solid surface, it can display different wetting behaviours, depending on the surface chemistry (i.e., surface energy) and the surface roughness (i.e., the texture of the interface) [8]. The contact angle (θ or CA) describes the primary state of wetting, which occurs as a droplet is unable to completely spread on an ideal smooth surface [9]. The basic understanding of the contact angles gives us an overall idea about the affinity/repulsion of the liquid spreading on a surface and the surface itself. The higher the CA is, the less is the affinity between the liquid and the solid surface and the lower the contact between the two. A surface is considered superhydrophobic if it displays CA > 150°. Pertaining to mechanical heart valves, the affinity or repulsion of blood for BMHV could be understood by investigating the contact angle variation: a high contact angle implies a low affinity of blood for BMHV and vice-versa. This affinity/repulsion will further dictate the adsorption kinetics of the cells/proteins, which is found at the very beginning of the coagulation cascade, thus determining the BMVH thrombotic profile. Under ambient conditions (in air), BMHV occluders are intrinsically hydrophobic [7]. This peculiar feature is predominantly related to their chemical composition, thus to the material BMHV are made of (pyrolytic carbon), being On-X^®^ valve the most hydrophobic.

It has been observed that complex hierarchical surface micro or nanostructures further reduce the droplet (liquid)-surface interactions. In these wetting patterns, the droplet sits on the top of microscale features such as tiny air pockets, resulting in a reduced liquid-solid contact [10, 11]. As a consequence the droplet exhibits an increased mobility, bouncing and even rolling-off on such textured surfaces [12, 13]. In other words, by controlling the surface roughness of mechanical heart valves, we can dictate the amount of interactions between blood and the valve itself.

As surface micro-texture represents a fundamental prerequisite to achieve low wettability substrates and may possibly improve thromboresistance, in this work, we aimed to create a tailored nano-texture by ultra-short pulse laser [14]. Ultra-short pulse laser processes enable to tailor surface textures of multiple scales from micro to nanometer range and to control their properties [15]. The laser textured occluders showed high water repellency, thus the super-hydrophobicity. Bark et al. [8], in their pioneering research showed that the superhydrophobic coating was able to dramatically reduce blood cell adhesion in a static environment compared to a bare pyrolytic carbon, demonstrating a reduction in the risk for thrombosis based on blood-material interactions.

In spite of all the progress made to date, it still remains controversial whether or not BMHV are thromboresistant and what factors the potential thromboresistance relies on. Albeit certain mechanical valves have been specifically developed to improve the thromboresistance, it is still debated whether this improved thromboresistance can be attributed to the design or to the material related features [16, 17]. Considering the importance of the subject, we aimed to address this topic from new perspectives, investigating the wetting phenomena of standard and laser-textured BMHV occluders in an under-liquid conditions. It is important to mention that investigation into the under-liquid wetting dynamics of BMHV has not been attempted. Considering the fact that operating condition of BMHV is in under-liquid arrangement, it is crucial to understand the wetting behaviour of heart valve prosthesis in such conditions. We believe the under-liquid wettability of pyrolytic carbon (PyC) mechanical valves will shed light into the mechanisms involved in their thromboresistance.

## Methods

The methodology of the present work is divided into two parts: materials and methods. In the materials section we discuss the types of BMHV used and how the micro-textures are introduced on the valves. In the methods section we discuss about the experimental methodology adopted for under-liquid wetting characterisation of the various BMHVs’ used.

### Materials

**Fig. 2:**
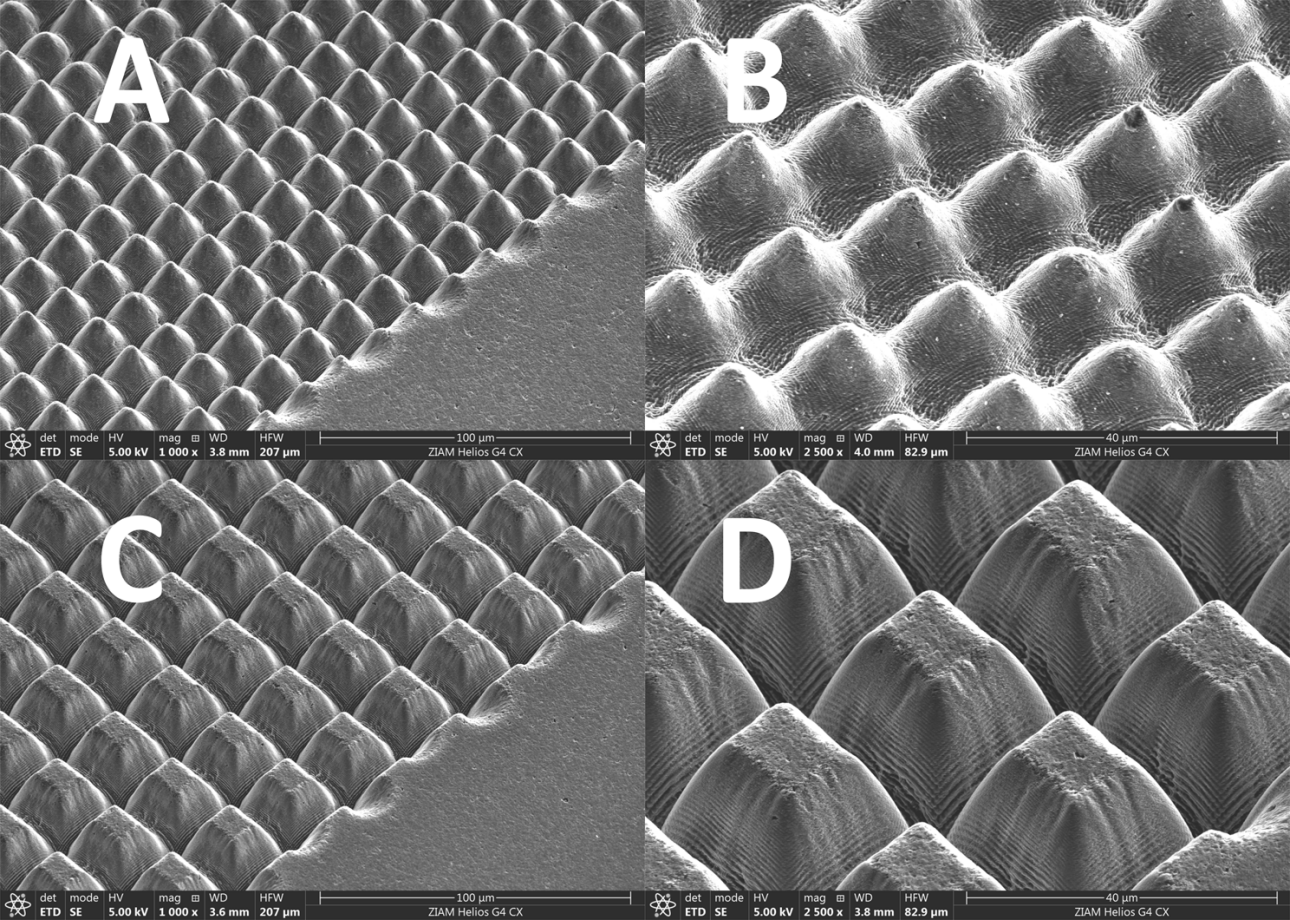
Laser texturing of the St. Jude Medical Regent^™^ showing the various “egg-box” patterns at different magnifications. **A**. The rounded shaped pillars, of an actual size of 16 µm (pitch) and 10,2µm (groove depth), observed at 1000x magnification. **B**. The same textures seen at 2500x magnification. **C**. The flat-top pillars of an actual size of 26 µm (pitch) and 14,6µm (groove depth) observed at 1000x magnification. **D**. The same textures seen at 2500x magnification.

Three pyrolytic carbon (PyC) occluders of two standard aortic St. Jude Medical Regent^™^ (St Jude Medical Inc., Minneapolis, MN, USA) 27- and 25-mm prostheses were irradiated by femto-second ultra-short pulsed laser (spot size of 20 μm and wavelength of 532 nm). Prosthesis selection was merely based on specific valve technical features of the housing, that made the disassembling and the reassembling of the occluders easier and on the availability of the same. In this preliminary phase of research, we only used the occluders, to simplify the laser texturing of the surfaces and to easily perform the wetting tests. The selection of the desired functional texture was certainly demanding. As little or nothing is known in this field, we arbitrarily employed “egg-box” configurations for our purposes, in order to take advantage of the simplest, most reproducible and tunable biomimetic geometry available. The “egg-box”-like configurations were made by over-scanning fixed cross-hatch patterns of single lines. The surface morphology of textured surfaces were strictly related to the laser power and the pitch. Pitch variation was arbitrarily set on 20 μm and 30 μm, where the former resulted in a well-rounded top and the latter in a flat surface on the top. As the configuration geometries were crucial in order to modify the liquid-solid interaction and in the absence of data in this specific field, we allowed a little variation in width and depth of the pillars, in order to obtain three different configurations (see Fig. 2). The laser texturing was performed by Lightmotif B.V. (Enschede, The Netherlands), a spin-off company of the University of Twente and the Materials Innovation Institute (M2i).

**Fig. 3:**
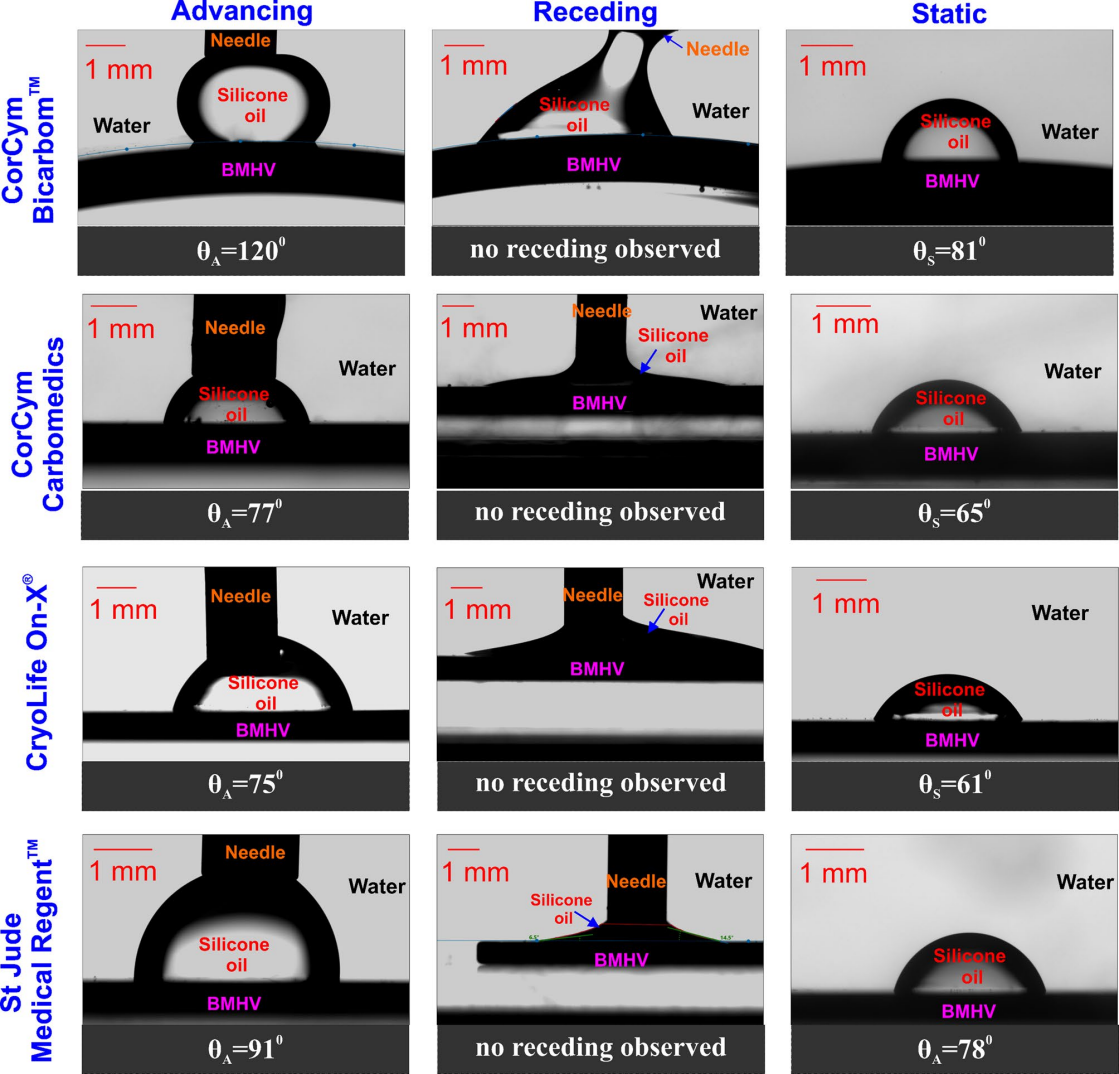
Underwater wetting measurement of four standard bileaflet mechanical valve occluders: Corcym, Carbomedics^™^ (Corcym SRL), St Jude Medical Regent^™^ (St Jude Medical, Inc, St Paul, Minn), Corcym, Bicarbon (Corcym SRL), On-X^®^ (On-X^®^ Life Technologies, Inc.). Silicon oil is used as the interacting droplet, while water mimicking blood is used as the surrounding fluid. θ_A: Advancing contact angle, θ_R: Receding contact angle, θ_S: Static contact angle

Further, four occluders of commercially available, standard PyC BMHV [1. Corcym Carbomedics^™^ (Corcym SRL), 2. St Jude Medical Regent^™^ (St Jude Medical, Inc, St Paul, Minn), 3. Corcym Bicarbon (Corcym SRL), 4. On-X^®^ (On-X^®^ Life Technologies, Inc.)] were also tested under the same conditions (see Fig. 3). Also, in this case, just the occluders of the valves were used for experiments, for the same reasons previously explained.

### Methods

In the under-liquid arrangement, Silicon oil (Sigma-Aldrich, Canada), was used as the droplet phase, while pure de-ionized water (purified by Milli-Q, Millipore Sigma, Ontario, Canada) was used as the surrounding phase, essentially to mimic blood. Silicon oil was used for the submerged wetting experiments, owing to the fact that any liquid soluble in water (such as blood) would have prevent the analysis of the droplet and the measurement of the CA’s. Therefore, it was necessary to use a liquid not soluble in water, silicon oil indeed, in order to prevent the drop from dissolving in water. Although water and blood have slight variation in the magnitude of density, viscosity and surface tension, both are considered polar fluids [18]. As a result, the nature of the attraction towards a polar surface or a repulsion from a non-polar surface remains consistent for both blood and water. While there may be a difference in magnitude of the contact angle, the wettability behaviour of blood is akin to that of water [18]. The properties of silicon oil and DI-water is appropriately mentioned in Table 1.

**Table 1:** Properties of the various fluids used in the present study

Fluid	Density (kg/m^3^)	Viscosity (mPa-s)	Surface tension (mN/m)	Interfacial tension-oil-water (mN/m)
Silicon oil	969	484	21	49
DI-Water	1000	1	72	-

We use optical goniometer (DSA 30, KRUSS, USA) for wettability characterization of the various BMHVs’. Wetting property is assessed through the measurement of contact angle (CA), where a higher CA (up to 180°), indicates stronger repellency between the solid and the liquid, essentially signifying a low chemical affinity or a phobic nature and vice-versa. Experiments were carried out in two specific arrangements of surrounding fluid: one in air and the other in water. Our main focus here is to understand the wetting dynamics in under-liquid arrangement (i.e., when water is the surrounding fluid), specifically to mimic the operational conditions of heart valve prosthesis. Experiments conducted with air as the surrounding fluid serve to establish correlations with the changes observed in the under-liquid arrangement.

To analyze the wetting properties, the static and dynamic contact angles (advancing and receding) of the various BMHV were carried out for oil (drop) in a water medium arrangement. For the measurement of static contact angle, a known volume of silicon oil is dispensed onto the substrate (the BMHV, which is kept in an under-liquid configuration), and the images are recorded (from the side) to obtain the contact angle. To measure the advancing contact angle, silicon oil (30 mL) was injected onto the substrate at a rate of 0.1 mL/s and as the contact line advances, the value of the contact angle is recorded. Similarly, for the measurement of the receding contact angle, the liquid was aspirated from the droplet at a rate of 0.1 mL/s. The receding contact angle is measured as the contact line of the droplet recedes. All Images are recorded at a rate of 50 frames per second with a resolution of 1280 × 1280 pixels^2^. For each image, a tangent is fitted to the three-phase contact line of the droplet to measure the appropriate contact angle.

We may mention that the hierarchical structures created by laser treatment results in superoleophobic wetting interactions on the occluders, as will be appropriately discussed in the result section. Thus, the force of adhesion of the silicon oil with the textured occluders is very low (in an under-liquid arrangement). Furthermore, in the underwater configuration (i.e., silicon oil is surrounded by water), the density contrast between silicon oil (density: 969 Kg/m3) and water (density: 1000 Kg/m3) results in a buoyancy force consistently acting in the upward direction. In a standard setup with the BMHV positioned at the bottom of the glass cuvette and surrounded by water, introducing a silicon droplet from the top (attached to a needle) and make it wet the BMHV, is not feasible as the force of adhesion is low compared to the buoyancy force. To address this, we use a J-type needle arrangement. In this setup, the substrate is fixed to the side walls of the glass cuvette at the liquid-air interface with the help of clips, and the droplet is dispensed in an inverted arrangement using a J-type needle. In other words, the silicon oil interacts with laser treated occluders in an inverted arrangement, in underwater situation.

All experiments were carried out at an controlled ambient temperature of 21^0^ C and relative humidity of 60%. Moreover, each individual experiments were repeated three times to ensure repeatability.

## Results

As can be observed from Fig. 3, in underwater conditions, all the standard BMHV PyC occluders (precisely the untreated BMHV occluders) showed very low contact angles (CA) with regard to oil, reflecting a pronounced affinity (philia: CA < 90˚) for nonpolar molecules (oil). No receding phenomena could be observed for the untreated BMHV, implying relatively strong chemical attractive forces specific to oil. Moreover, the smallest static contact angle of around 61˚ could be observed for the untreated CryoLife On-X^®^ valve, the only valve made of full PyC (see Fig. 3).

**Fig. 4:**
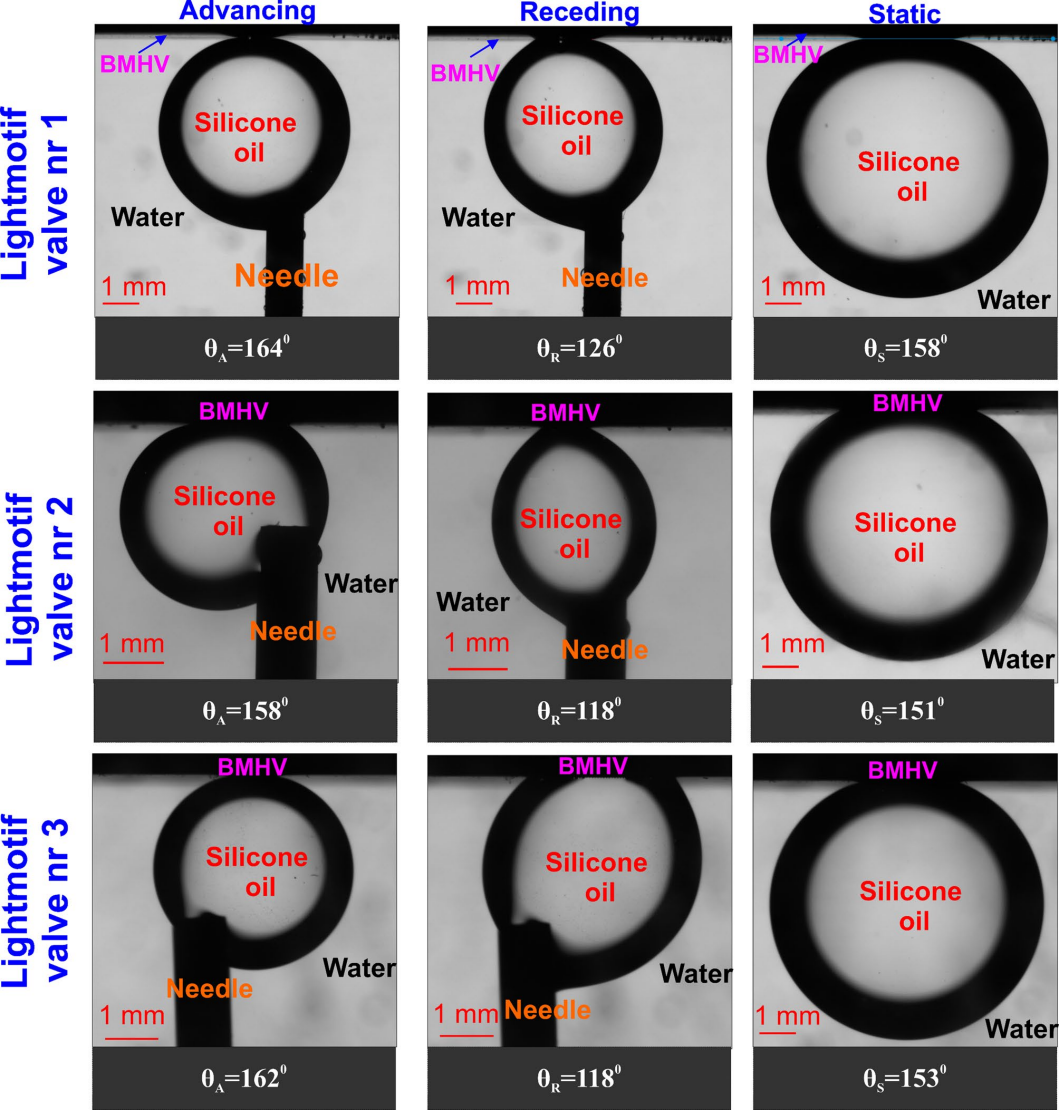
Underwater wetting measurement of three laser treated mechanical valve pyrolytic carbon occluders: Lightmotif valve nr 1, Lightmotif valve nr 2, Lightmotif valve nr 3. Silicon oil is used as the interacting droplet, while water mimicking blood is used as the surrounding fluid. θ_A: Advancing contact angle, θ_R: Receding contact angle, θ_S: Static contact angle

On the other hand, the laser-treated PyC occluders strongly repelled oil in underwater conditions (superoleophobic; CA > 150˚). A drastic change in their wetting behaviour was observed, depending on the surrounding fluid. As such, when the surrounding fluid was air, the laser treated PyC occluders showed hydrophobic nature with CA of around ∽ 112˚ (for water/blood), thereby implying a water/blood repellency. While on changing the surrounding fluid to water (i.e., in underliquid arrangement), the same occluder showed a oleophobic nature (Fig. 4).

## Discussion

The aim of the present study was to address the following questions: are mechanical valves truly thromboresistant, as they require lifelong anticoagulation? Given the fact that the two concepts are diametrically opposed, could mechanical valves be thromboresistant and thrombogenic at the same time? What does the thromboresistance of mechanical valves mostly depend on?

The thrombogenicity of mechanical valves has already been the subject of extensive research and involves the well-known “Virchow’s triad”, where blood, surface, and flow represent the three key factors in the so-called blood–artificial surface interaction [19]. Given the complexity of the topic, we preferred to focus on the surface of the PyC occluders, primarily investigating the two aspects involved in the liquid-solid or blood-artificial surface interactions: the surface roughness (interface) and the surface energy. A comprehensive grasp of these mechanisms is a fundamental prerequisite for understanding the inherent characteristics of mechanical valves and for developing innovative surface modification. S. Forti et al. analyzed the role of the chemical and morphological characteristics in protein adsorption and platelets binding of biomaterials, one of which was PyC [20]. PyC resulted as the lowest adhesive surface. Concerning the extent of platelet activation, platelets adherent to PyC were mostly weakly activated. Pyc showed to have a good hemocompatibility, besides anti-thrombogenic properties [20]. Interestingly, PyC resulted one of the most hydrophilic biomaterial (in atmospheric conditions), with a CA mean value of 58.0˚ and the one that induced the lowest level of total protein adsorption and platelet adhesion. The topic is still controversial in literature [21], since it is likely that differences in chemical and morphological surface composition may account for an inverse behavior of protein adsorption when analyzed as a function of contact angle measurements.

We delve deep into the topic, analyzing the wetting behaviours of unmodified mechanical valve occluders and of “textured” hydrophobic occluders in underwater conditions. The presence of a nanometric texture (for the Laser-treated surfaces) on the surface of PyC occluders was responsible of the change in their wettability behaviour. This observation is contrary to the standard notion that a hydrophobic surface should demonstrate an oleophilic nature, due to its high affinity towards non-polar molecules. Since no such observation was seen for the laser-treated PyC, this imply that the surface nano-textures are playing a crucial role on the overall wettability of the substrate.

The nanostructure ensures that a thin-layer of surrounding fluid (air and/or water) limits the contact of droplet (oil) with the surface. Due to this fact i.e., the droplet sliding on the thin-layer of water, a constant contact angle hysteresis could be observed for all the laser-treated MHVs’ (Fig. 4). But as per intuition, the presence of thin layer should ensure that the droplet exhibit a spherical shape (i.e., CA ∽ 180˚), since the droplet does not contact the surface at all. However, as observed in our results, the contact angle measured on the laser treated occluders was < 180˚ (Fig. 4). This imply that the thin layer might have ruptured at certain points, thereby ensuring the droplet contacts the surface at those points. The presence of the thin layer between the droplet and the surface and its rupturing at certain contact point (in an under-liquid arrangement) was also observed by Daniel et al. [22]. Thus, from a pathophysiological perspective (“in-vivo”), it may be argued that a textured BMHV even might promote thrombosis, as the nanometric roughness of the surface limits the intrinsic advantage of a standard PyC BMHV, with a strong affinity for non-polar molecules [23–27]. In a “in-vivo” scenario, the nanostructure will ensure that a thin blood film permanently remains in contact with BMHV occluders, dramatically increasing the thrombogenicity. While our experiments did observe thin film rupture, achieving a complete rupture could limit the blood contact with the laser treated occluders, potentially making them suitable for thromboresistance. However, it is essential to consider that thin film rupture process depends on numerous factors and is a time-consuming phenomena, which may not be advisable for in-situ medical devices. Therefore, the functionality of superhydrophobic surfaces (i.e., nanostructured surfaces) ends up being as a limitation for in-situ medical devices. As it is expected in such devices that the surface has limited contact with blood (the surrounding fluid), which is not the case here. On the other hand, the standard PyC occluders, submerged in a polar fluid, surprisingly exhibited a greater affinity for non-polar fluids, particularly if made of pure pyrolytic carbon. This phenomenon allows the formation of a thin, but consistent surface layer of non-polar molecules, on which polar fluids (such as blood) can slip off or at least where the contact between the solid surface and the surrounding liquid is prevented.

### Limitations of the study

The present study has a few theoretical and empirical limitations, strictly related to the kind and the topic of the research. The primary endpoint of the present study was to shed light on BMVHs thrombogenicity and thromboresistance, and to allow a significant breakthrough in a field, that has remained at a standstill for a long time. We addressed BMVH thrombogenicity from novel perspectives, never investigated before, showing new paradigms assuming that the blood-surface interaction is primarily a liquid-solid wetting interaction. The topic becomes even more stimulating, considering that the aim of the study was to perform the experiments in underwater conditions. These conditions are in general very critical and complex, but they become even more demanding given the peculiar nature of the elements involved, such as blood. If on one hand this context has certainly represented a fascinating challenge, on the other hands all these factors together definitely constituted a limiting factor. The tests were performed in a static environment, whereas “in vivo” these same conditions are of unsteady nature. Besides, the costs of the heart valve prosthesis, as well as of the laser treatment certainly impacted the study.

Moreover, in the wetting experiments, water was used instead of blood because of their similar wetting behaviours and its ease of use. According to literature and to our previous experiments, water and blood deposited on PyC BMHV occluders spread in a similar way, displaying comparable contact angles. Mimicking water instead of blood poses inherent challenges in precisely mirroring the intricacies of “in-vivo” scenarios. However, it should also be mentioned that in our previous experiments we observed that blood droplet shows a certain instability and a propensity to spontaneously migrate, making the measurements critical and inconsistent. This event was also observed even after warming the blood approximately up to the body temperature of ∽ 36.0 °C, adding Heparin (1 cc or 5.000IU) to the sample and/or using dedicated Teflon coated needles.

Additionally, aspects such as the protein adsorption kinetics, and fluid-BMHV interface turbulence demands a separate study altogether. In spite of all the limitations, we believe the results observed underpins the critical role of surface energy on the thrombosis of BMHVs’, thereby paving a new direction for future endeavours.

## Conclusions

The above-mentioned observations and findings allowed us to draw the following conclusions. Currently available standard BMHV “fear” water and blood. The intrinsic affinity of standard BMHV for nonpolar fluids can be translated into a tendency to repel fluids with polar molecules, such as blood. The blood-artificial surface interaction in standard BMHV is effectively minimized. The contact between blood and standard BMHV is drastically reduced by polar-non-polar Van der Waals forces. The “hydro-bloodphobia” of standard BMHV is intrinsically related to their chemical composition and their surface energy. The surface roughness or interface does not play a significant role. The thromboresistance of standard BMHV lies in their intrinsic ability to repel water and blood. This peculiar feature lies in the material, BMHV are made of: pyrolytic carbon indeed. The wettability of standard BMHV can be tuned, varying the surface interface, by means of nanotechnology.

### Electronic supplementary material

Below is the link to the electronic supplementary material.

Supplementary Material 1

## Abbreviations

BHV: Biological Heart Valve Prostheses
BMHV: Bileaflet Mechanical Heart Valve Prostheses
CA: Contact Angle
MHV: Mechanical Heart Valve Prostheses
PyC: Pyrolytic Carbon
SJM: St. Jude Medical Regent^™^
